# Early Signs of Memory Impairment among Multiple Sclerosis Patients with Clinically Isolated Syndrome

**DOI:** 10.3233/BEN-2012-110201

**Published:** 2012-02-15

**Authors:** Theodora Panou, Vasileios Mastorodemos, Efrosyni Papadaki, Panagiotis G. Simos, Andreas Plaitakis

**Affiliations:** ^1^Department of NeurologyFaculty of MedicineUniversity of CreteHeraklionGreece; ^2^Department of PsychologyUniversity of CreteRethymnoGreece; ^3^Department of RadiologyFaculty of MedicineUniversity of CreteHeraklionGreece

**Keywords:** Multiple sclerosis, memory, executive function

## Abstract

The study investigates primary and secondary verbal memory and motor/executive functions (response inhibition and strategy shifting ability) in multiple sclerosis (MS) patients with clinically isolated syndrome (CIS). We studied 44 CIS patients and compared them to 49 patients with relapsing remitting MS (RR-MS) displaying mild disability and to a large cohort of age- and education level-matched healthy volunteers (*n* = 230). Results showed that both CIS and RR-MS patients evidenced a disproportionate impairment in the immediate and delayed recall of the second (as compared to the first) of two short narratives of the Logical Memory WMS-III subtest, and reduced performance on the Memory for Digits-Forward. Performance of either group on the executive tasks was not impaired, showing evidence of a reversed speed-accuracy trade-off. Illness duration emerged as a significant predictor of memory and executive task performance. Clinical, psychoemotional, and brain imaging findings were also examined as potential correlates of memory deficits and disease progression among CIS patients. These findings may signify early-onset decline of specific cognitive functions in CIS, which merits regular follow-up assessments and monitoring of psychoemotional adaptation and everyday functioning.

## 1. Introduction

Multiple Sclerosis is considered to be an autoimmune disorder of the CNS typified by inflammatory demyelination and secondary axonal degeneration. While lesion/symptom dissemination in space and time is a characteristic feature of MS, the disorder often starts as a clinically isolated syndrome (CIS). The diagnosis of CIS is given to patients who have experienced a single episode of neurological involvement, such as optic neuritis, brainstem or spinal cord dysfunction. While CIS often represents the first episode of MS, the term is, also, used to define a monosymptomatic or monophasic inflammatory demyelinating CNS disease that may or may not progress to MS [51]. Multisymptomatic presentations, indicative of dissemination in space, are associated with a higher conversion rate to clinically definite MS [42]. In this regard, brain imaging findings appear to have a predictive value. Thus, while CIS patients without CNS lesions on MRI are not likely to convert to clinically definite MS [61], the majority of those with MRI-visible brain lesions are expected to do so [4,6]. Moreover, there is evidence that the number and distribution of MRI lesions, detected at the time of the initial clinical presentation, is predictive of MS course, including the degree of subsequent disability [43,44,46]. In contrast, other studies maintain that, while the presence of MRI lesions predicts the devel-

ISSN 0953-4180/12/$27.50 © 2012 – IOS Press and the authors. All rights reserved opment of multiple sclerosis, there is a limited relationship between lesion accumulation and subsequent physical disability.

Whereas the role of baseline MRI findings and focal neurological deficits in determining the risk of developing clinically definite MS has been studied extensively, the predictive value of cognitive involvement has not been adequately investigated. In particular, the cognitive performance of MS patients in the very early phases of the disorder has been studied sparsely. Moreover, results obtained have been somewhat variable, with the prevalence of cognitive impairment detected in CIS ranging from 25% to 57% [18,24,53,67]. Few studies on CIS patients have explored the nature of their neuropsychological impairment. This has included verbal short-term and working memory, speed of information processing, and, secondarily, executive function and attention [18,24,37]. Some researchers have suggested that the most frequently affected domain is attention and concentration along with verbal fluency [1,8,16]. It is presently unclear whether the detected variability in performance relates to clinical features, such as mode of clinical presentation, disease progression and physical disability or to imaging findings [26,50]. Also, methodological differences, particularly pertaining to the criteria used for impaired performance and the lack of age and education adjusted norms, may explain some of the controversial results.

The primary goal of the present study is to assess neuropsychological and psychoemotional features, focusing on primary and secondary episodic memory, in patients with CIS. In addition, measures of executive function, namely inhibition and task-switching ability, were assessed controlling for the potential effects of the disorder on processing speed. Specifically, the performance of a group of 44 CIS patients on these measures was compared to normative data with respect to (a) mean scores and (b) the rates of single and/or multiple cognitive deficits normally encountered among age- and education level-matched neurologically intact adults ($n = 230$). In addition, potential clinical, cognitive and psychoemotional predictors of disease progression were studied among 25 CIS patients who were followed for up to three years after disease onset. We also evaluated brain imaging findings (T2 MRI data) of CIS patients regarding the location of demyelinating lesions and how these correlated with memory performance of these patients and/or with disease severity and progression. Lastly, the performance of the CIS group was compared to the scores of 49 patients with established Relapsing-Remitting MS (RR-MS), who experienced very mild functional disability. The latter were matched on demographic, psychoemotional and IQ variables to the group of CIS patients.

## 2. Method and procedure

### *2.1. Participants*

The target patient group comprised 44 patients who presented clinically with a single episode with symptoms typical for MS [54] and who had one or more MRI showing lesion(s) suggestive of demyelinating disease ([3]; incorporated into the McDonald criteria). The CIS patients were consecutively referred to the Neurology Department at the University Hospital of Heraklion over a 3-year period (January 2007–December 2009). Follow up clinical and MRI data were available on all CIS patients for up to three years after the neuropsychological evaluation (median = 2.5 years). In these patients the disease had run a course ranging from 1–12 years post disease onset (median = 4.0 years). Disease progression was based either on clinical evidence requiring the occurrence of a second clinical episode documented by objective new neurological signs or on the appearance of additional CNS lesion(s) in MRI, thus documenting dissemination in time and space (McDonald's criteria for Clinically Definite MS). Although CSF studies are not required for diagnosis according to the currently used criteria (McDonald, original and revised), all but one CIS patients underwent lumbar puncture. However for 11 CIS pts data on the presence of oligoclonal bands or increased IgG index are not applicable. Ten CIS patients (7F, 3 M) had increased IgG index. The remaining 22 (6F, 16M) CIS patients had a negative lumbar puncture. Only 7 CIS patients were at the time of the cognitive assessment under treatment with immunomodulatory agents (interferon). Detailed clinical information is presented in the Results section.

The severity of potential cognitive impairment among CIS patients was evaluated (a) through comparison with normative data on a series of neuropsychological tests with a focus on memory (and examination of age- and education-adjusted standard scores) and (b) by comparison with performance of a selected group of patients ($N = 49$) who had received a definite diagnosis of MS with a relapsing-remitting course (RR-MS) but showed relatively mild functional impairment as defined by Expanded Disability Status Scale (EDSS) scores of $\leqslant 3$ [33]. MS patients were recruited through the same institution as CIS patients as previously described [38]. The diagnosis of MS or CIS was established by a Board Certified Neurologist (AP or VM) using the clinical and MRI criteria of the International Panel on MS [41]. Onset of disease was defined as the year the patient first experienced clear symptoms of MS as defined by Poser [54]. All symptoms experienced within three months after the first symptoms were regarded as symptoms at onset. Twenty-six RR-MS patients (as compared to only 7 CIS patients) were on treatment with immunomodulatory agents (interferon, glatiramer, or natalizumab). When tested, RR-MS patients were in remission for at least 30 days following their last relapse. A relapse (exacerbation) was defined as the appearance of new neurological symptoms or a worsening of pre-existing symptoms, lasting at least 24 h and preceded by a period of clinical stability or improvement of at least 45 days. Fatigue or transient fever-related worsening of symptoms were not considered as relapses. Since MS relapses are conventionally treated with corticosteroids given intravenously for 3 days, followed by oral tapering lasting for 12 additional days, the patients were examined at least 30 days after the last steroid treatment.

Additional inclusion criteria for both clinical groups included: (a) negative history of alcohol or drug abuse, head injury with loss of consciousness, schizophrenia or bipolar disorder, learning disability or any neurological disorder other than MS, (b) absence of significant visual or motor impairment that would interfere with cognitive testing, and (c) absence of other autoimmune, immune-mediated and infectious diseases of the CNS as evaluated by means of neurological history and examination, laboratory tests and neuroimaging studies. As described in more detail below, the two groups were matched for age, education level, estimated IQ, and presence of symptoms indicative of mood or anxiety disorder. The median time post disease onset at the time of the neuropsychological evaluation was 1.5 years for the entire groups of CIS patients and 6.5 years for RR-MS patients. The study was approved by the Herakleion University Hospital Ethics Committee and written informed consent was obtained from all patients after being briefed on the details of the study.

### 2.2. *Neuropsychological measures*

Participants received a battery of cognitive tests administered by the same clinical neuropsychologist over one session. Given that several tests routinely used to assess global cognitive function in MS are not available in Greek, and the main focus of the present study was on memory function, a customized battery of tests was employed to address specific hypotheses regarding the potential source of memory problems. Two subscales from the Wechsler Abbreviated Scales of Intelligence (WASI; [64]) were used as indices of crystallized (Vocabulary) or fluid intelligence (Matrices).[1] Immediate verbal memory was assessed with the Memory for Digits subtest from the Wechsler Memory Scale (WMS-III; [63]). Secondary verbal episodic memory was assessed with the WMS-III Logical Memory subscale (WMS-LM) and secondary non-verbal (visual) episodic memory with the Taylor Complex Figure. The following indices were computed from WMS-LM: (i) Number of elements correctly recalled from Story A in the immediate recall condition (LM-I-A), (ii) Number of elements correctly recalled from Story B-immediate recall on the first trial (LM-I-B1), (iii) Number of elements correctly recalled from Story B-Immediate recall on the second trial (LM-I-B2), (iv) Immediate Recall Index ([i] plus [ii]), (v) Number of elements correctly recalled from Story A after a 30 min delay without reminding (LM-II-A), (vi) Number of elements correctly recalled from Story B after a 30 min delay without reminding (LM-II-B), (vii) Delayed Recall Index ([v] plus [vi]), (vii) LM Retention Index-Story A ([v] / [i]) * 100, (viii) LM Retention Index-Story B ([vi] / [iii]) * 100, (ix) Learning Slope ([iii] minus [ii]), (x) LM Recognition index (number of items correctly recognized following a 30 min delay from both stories, (xi) Differential Immediate Story Recall Index ([i] minus [ii], and (xii) Differential Delayed Story Recall Index ([v] minus [vi]. Separate indices for Story A and Story B as well as differential-story recall indices were considered based on a previous report of increased difficulty among RR-MS patients in memorizing Story B [48]. Primary and working verbal memory were assessed through the Memory for Digits WMS-III subtests (Digits Forward and Digits Reverse scores).

The modified Taylor Complex Figure test (TCF; [27]) was used to obtain a measure of secondary, visuospatial episodic memory. Three scores were derived: (a) Copying (number of elements correctly copied from the figure), (b) Delayed Reproduction (number of elements correctly copied from the figure after a 3–5 minute delay), and (c) TCF Retention index ([b] / [a]) * 100 (a ratio method was chosen rather than the

[1] Standardization of the WASI in the Greek population is under way by another group [40]. WASI and WMS-III subtests used in this report were adapted for research purposes with permission from NCS Pearson, Inc., San Antonio, TX.

customary calculation of a difference score to facilitate comparison between TCF and LM-based retention indices).

Patients were also tested on two computerized tasks designed to assess specific executive skills. The Inhibition task assessed the ability to inhibit a learned manual stimulus-response (S-R) association and adopt a novel S-R association. During each block of trials one of two capital letters were flashed at the center of the computer screen at a rate of 1 letter every 1.5 sec and participants had to press the key corresponding to the letter they saw each time. Following a series of 6 learning/habituation trials (Baseline Condition) the patients were asked to reverse their response strategy and press the "non-corresponding" key for the next 14 trials (Change Trials). For example, they were first asked to press A if they saw the letter A and L if they saw the letter L. After the 6th trial they were asked to start pressing the A key in response to the letter L and the L key in response to the letter A. A different pair of randomly arranges letters was used on each of the four blocks of 20 trials. Number of errors and RT for correct responses was stored electronically and used to compute the following performance indices: (i) average RT across all Baseline trials, (ii) average RT across all Change trials, (iii) Mean error rate for Baseline trials, (iv) Mean error rate for Change trials, (v) Inhibition Index-RT representing the proportional increase in mean RT between Baseline and Change trials, and (vi) Inhibition Index-Errors representing the proportional increase in average error rate between Baseline and Change trials. Only trials with RTs between 200 and 5000 ms were included in the computation of the aforementioned indices to eliminate outlier responses.

The Strategy Set-Shifting Task was designed to assess the ability to alternate between two simple cognitive strategies depending on the modality of the stimulus. Auditory (animal names) and visual stimuli (drawings of the same animals in various-single-colours) were presented in a pseudorandom order. Target stimuli, requiring a right mouse-key press were names of four-legged animals and red animal drawings. All other stimuli required a left key press. The order of stimuli was designed to afford computation of mean RT and error rates for each of three experimental conditions: (a) Constant Trials (CT) which were always preceded by a same-modality stimulus and did not require a shift in either cognitive strategy or responding hand (e.g., /Visual-Target/, /Visual-Target/, /Visual-Target/ [CT trials are underlined]); (b) Response-Switch Trials (RST) which required only a switch in responding hand (/Auditory-Target/, /Auditory-NonTarget/, /Auditory-Target/ [RST trials underlined]), and (c) Strategy & Response Switch Trials (SRST) requiring a switch in both strategy and responding hand (/Visual-Target/, /Auditory-Non-target/, /Visual-Target/ [SRST trials underlined]). The following indices of set-shifting ability, controlling for overall processing speed, were computed: (i) Strategy & Response Switching indices (mean RT and error rate difference between SRST trials and CT trials), and (ii) Response Switching indices (mean RT and error rate difference between RST trials and CT trials).

Normative data from the Greek population were available on each measure were available from a sample of 550 native Greek individuals aged 16–60 years stratified by educational level and geographic origin (cities with population over 10,000: 54%, towns with population ranging between 2,000–5,000: 29%, and towns with population under 2,000: 17%; [58,59]). This approach permitted computation of age- and education-adjusted standard scores, separately for six subgroups defined by age (16–38 and 39–60 years) and education level (0–9, 10–12, and 13+ years of formal education). Subgroup size ranged between 50 and 100 persons.

Two self-report scales were administered to all patients assessing depression and anxiety symptomatology: (a) The Greek adaptation of the Center for Epidemiology Studies Depression Scale (CESD; [22,56], with a score of 24/60 and higher indicating the presence of clinically significant depression, and (b) The State-Trait Anxiety Inventory Form Y [23,60]. In the normative data reported by Fountoulakis et al. [23] mean ($\pm$ SD) scores were 25 $\pm$ 11 for STAI-A and 28 $\pm$ 11 for STAI-B. Finally, the Fatigue Severity scale (composed of 9 statements associated with a 7-point scale) was administered to all patients in order to assess the severity, frequency and impact of fatigue on daily life [32]. A score of 36 or higher has been proposed as indicative of clinically significant perceived fatigue levels [20].

### *2.3. Magnetic resonance imaging acquisition and analysis*

All MS and CIS patients underwent a brain MRI in a 1.5 T MR scanner at least annually using a protocol dedicated to MS. This comprised of a T1 spin echo sequence (TR/TE: 600/15 msec) before and after intravenous Gadolinium administration in the axial and coronal planes, a T2 turbo spin echo sequence (TR/TE: 5000/98 msec) in the axial plane and a turbo FLAIR sequence (TR/TE/TI: 9000/120/2600 msec) in both axial and sagittal planes. All scans were interpreted by two experienced neuroradiologists. Demyelinating lesions were identified on T2 sequences as hyperintense foci and classified according to their location under the following sectors: periventricular white matter, corona radiate, centrum semiovale, subcortical white matter, basal ganglia, thalami, corpus callosum, cerebellum, midbrain, cerebellar peduncles, and medulla. Lesions detected were also classified according to their enhancement on post-Gadolinium T1 sequences. In addition, the total number of supratentorial and infratentorial lesions was measured. We analyzed the MRI data of the CIS group only, as the main focus of the study was to study potential cognitive deficits among CIS patients.

### 2.4. Analyses

The distribution of individual subtest, composite, and index scores in the normative sample for all cognitive tests used in the present study approached the normal distribution in all cases (as indicated by skewness and curtosis values ranging between $-0.5$ and $+0.5$). Accordingly, it was appropriate to convert patient raw scores to z scores. Analyses comparing patient groups were also performed on raw scores (given that the two groups were matched on age and education level), but in all cases results were identical to those obtained on the basis of standard scores, which are reported here to facilitate comparison with other studies.

Initially, mostly descriptive analyses were performed in order to characterize the general cognitive and psychoemotional profile of the present group of CIS patients through comparisons with published normative data and with the scores of patients in the RR-MS group.

Next, the first primary goal of the study was addressed by contrasting the performance of patients in the CIS group to normative population estimates adjusted for age and education level. This was achieved through (a) one-sample t-tests on patient standard test scores and (b) Chi-square tests comparing the proportions of CIS patients who scored in the impaired range (as defined by $z < -1.5$) to the corresponding proportions observed in the normative sample. Similar comparisons were performed between CIS and RR-MS patients. These analyses were supplemented by one-way ANOVAs with three levels on the group factor (Controls, CIS, RR-MS) on each dependent variable (raw scores). Significant F ratios (at $a = 0.002$) were followed up by Bonferroni-corrected pairwise comparisons. The control group consisted of 230 adults selected from the standardization sample, matched on age, education level and Verbal IQ with each of the clinical groups ($p > 0.5$ in each case).

For cognitive indices where CIS patients showed significantly reduced performance we, also, explored potential clinical (duration of illness, EDSS, number of neurological symptoms) and psychoemotional correlates (depression, anxiety, and fatigue scores) by computing Pearson or Spearman correlation coefficients. Additionally, the relation between cognitive status and clinical/psychoemotional variables was assessed at a group level by comparing subgroups of CIS patients with and without significantly impaired performance on one or more memory and executive indices.

The potential predictive value of cognitive and psychoemotional variables on disease progression was assessed by two complementary ways. First, by comparing the baseline performance of CIS patients who, at the two-to-three year follow up, satisfied the clinical and/or radiological criteria for clinically definite MS ($N = 25$), to the performance of CIS patients who did not satisfy these criteria. Second, by logistic regression analyses that explored the joint predictive value of these variables for disease outcome.

Finally, the association between lesion location (coded as a nominal variable) and neuropsychological performance (z scores) was assessed for CIS patients through a series of point-biserial correlations.

## 3. Results

### 3.1. Clinical and demographic data of both patients' groups

The CIS-patient group included 44 patients. Of these, 38 had monofocal and 6 multifocal disease. Regarding MS onset, 15 (34.1%) patients presented with optic neuritis, 10 (22%) with brain stem-cerebellar involvement, 7 (16%) with spinal cord dysfunction and 6 (14%) with sensorimotor (long tract) symptoms. The remaining 6 (14%) patients experienced a polysymptomatic onset (Table 1). These data are similar to those reported for other CIS cohorts [31]. All CIS patients included in this study showed white matter lesion(s) that could be attributed to MS ([3], incorporated into the McDonald criteria). The clinical characteristics of these patients are presented in Table 1.

As indicated above, all CIS and RR-MS patients included in this study had EDSS scores $\leqslant 3$ (range: 0–3). The proportion of patients with EDSS scores $\leqslant 2$,

Table 1
Clinical and demographic information on the patients and controls

| | RR-MS ($n = 49$) | CIS ($n = 44$) | CIS→CDMS ($n = 25$)[1] | Controls ($n = 230$) |
|---|---|---|---|---|
| Gender (M/F) | 20/29 | 20/24 | 13/12 | 103/127 |
| Handedness (R/L) | 47/2 | 41/3 | 23/2 | 217/13 |
| Education (years) | 15 ± 2.3 (9–16) | 15 ± 2.8 (6–18) | 15 ± 2.4 (9–18) | 14,4 ± 3,2 (6–19) |
| Current age (years) | 34,80 ± 7,3 (15–50) | 32,0 ± 9,5 (15–51) | 32,3 ± 9,1 (16–52) | 33,25 ± 9,9 (16–54) |
| Medication: | | | | |
| No treatment | 23 | 36 | 14 | – |
| INF or Glatiramer acetate | 23 | 7 | 11 | |
| Natalizumab | 3 | – | – | |
| First symptom, n (%): | | | | |
| Sensorimotor | 12 (24.5) | 6 (13.6) | 3 (12.0) | – |
| Optic neuritis | 10 (20.0) | 15 (34.1) | 7 (28.0) | |
| Brainstem-cerebellar | 10 (20.0) | 10 (22.7) | 6 (24.0) | |
| Spinal | 7 (14.3) | 7 (15.9) | 5 (20.0) | |
| Polysymptomatic | 10 (20.0) | 6 (13.6) | 4 (16.0) | |
| Age at disease onset: *mean* ± *SD* (range) in years | 29 ± 7.5 (14–48) | 30.2 ± 9.0 (14–49) | 30.8 ± 8.6 (14–49) | – |
| Illness duration[2]: *mean* ± *SD* (range) in years[†] | 6.5 ± 4.5 (1–17) | 1.9 ± 1.96 (0.1–7.5) | 1.5 ± 1.7 (0.1–6) | – |
| Number of relapses (median) | 2.6 ± 2.1 (1–9) | –[2] | 1.0[3] (0–3) | – |
| EDSS[2] (median)[†] | 2.0 ± 0.7 (0–3) | 1.0 ± 0.5 (0–2.5) | 1.0 (0–2.5) | – |
| Current EDSS (median)* | – | 1.0 ± 0.7 (0–3.5) | 1.0 (0–3.5) | – |

[†]Significant RR-MS vs. CIS group differences ($p < 0.0001$).
*Significant difference between CIS patients who maintained their status as of December 2010 and those who converted to CDMS ($p < 0.02$).
[1]Subgroup of CIS patients who, as of December 2010, fulfilled clinical and/or radiological criteria for Clinically Definite MS (baseline information is presented here).
[2]At the time of the neuropsychological evaluation.
[3]At the most recent clinical follow-up (December 2010).
Abbreviations: EDSS: Expanded Disability Status Scale.

indicating mild physical disability, was similar between the CIS (86.4%) and the present (selected) group of RR-MS patients (86.7%). Of the 49 RR-MS patients included in this study, 16 (28.6%) had MS for 10 or more years after onset. These patients were considered as suffering from benign MS as previously described (the patient's EDSS was < 3.0 10 or more years after onset) [38].

Follow-up neurological evaluations were performed within three years after the initial neuropsychological evaluation. Of the 44 CIS patients evaluated at that time, 16 evidenced a clinical relapse, whereas 9 showed CNS additional lesion(s) on MRI without having experienced such a relapse, thus fulfilling the criteria for clinically definite MS. The median interval from disease onset to conversion to CDMS was 2.3 years (IQR: 1.25–3.46, range 0.8–5.4 years) for those who had a clinical relapse and 1.5 years (IQR: 0.71–4.25, range 0.5–8.0 years) for those who fulfilled the radiological criteria. There was no systematic difference between the CIS patients who showed dissemination in time and space and those who experienced a clinical relapse on the delay between disease onset and neuropsychological evaluation (median time elapsed was 0.75 vs. 0.87 years). Neither gender nor clinical presentation at disease onset was significantly associated with rate of conversion to CDMS ($X^2$ tests, $p > 0.5$). There was, however, a tendency for higher conversion rate (67%) among CIS patients with polysymptomatic onset as compared to CIS patients who presented with optic neuritis (33%). Age, disease duration and EDSS at the time of neuropsychological assessment did not differ between those who converted to CDMS as compared to those who remained with clinically isolated syndrome. As expected, current EDSS (as of December 2010) was significantly higher for those who converted to CDMS (median = 1.50; range: 0.0–3.5) than for patients maintaining their CIS status (median = 1.0; range: 0.0–2.5, Mann-Whitney $z = -1.90, p = 0.03$). Additional clinical information on these patients is presented in the right-hand column of Table 1.

### 3.2. Memory, executive, and psychoemotional function of CIS and RR-MS patients

As shown in Table 2, both patient groups (CIS and RR-MS) had estimated general intelligence indices in the average of above range (as indicated by $z > -1.0$ corresponding to 85 points): however, they scored significantly higher than average on the verbal subtest (WASI Vocabulary) and slightly lower than average on the non-verbal subtest (WASI Matrices). The two groups did not differ on mean CESD, STAI-A, and STAI-B scores ($p > 0.2$), although the proportion of patients with scores indicative of symptoms of depression (as indicated by scores > 23; [22]) was significantly higher in the RR-MS than in CIS group (33% vs. 16%, $p = 0.05$). The proportions of patients with potentially clinically significant state (CIS: 26% vs. RR-MS: 42%) or trait anxiety (CIS: 51% vs. RR-MS: 58%), as indicated by scores at least 1.5 SD above the normative sample mean [23], were not significantly different between groups. It should be noted, however, that only one of the CIS patients carried a formal diagnosis of an anxiety and/or a mood disorder as compared to four RR-MS patients (two patients were treated with SSRIs at the time of testing). RR-MS patients reported higher levels of fatigue and impact on daily life, $F(1,84) = 5.56, p = 0.021$. Forty-four percent of patients in the CIS group reported potentially significantly elevated fatigue levels (indicated by a score > 36 on the Fatigue Severity Scale) as compared to 60% in the RR-MS group ($p > 0.5$).

Supplementary one-way ANOVAs with three levels on the group factor (Controls, CIS, RR-MS) on raw scores(evaluated at a = 0.002) indicated that overall group effects were more pronounced on Memory for Digits Forward, $F(2,320) = 6.76, p = 0.001$, Memory for Digits Reverse, $F(2,320) = 6.16, p = 0.002$, Immediate recall of WMS-III Story B (first trial), $F(2,320) = 12.83$, $p = 0.0001$, Delayed recall of WMS-III Story B, $F(2,320) = 5.77$, $p = 0.003$ (marginal), Immediate, $F(2,320) = 7.72$, $p = 0.001$, and Delayed Differential Story Recall Indices, $F(2,320) = 5.35$, $p = 0.005$ (marginal). These effects remained significant after controlling for individual differences in WASI Vocabulary. Significant main effects of Group were also noted for RTs on both conditions of the Inhibition task, $F(2,320) = 6.99, p = 0.002$ and $F(2,320) = 6.84$, $p = 0.002$, respectively, and on the Inhibition Index, $F(2,320) = 7.09$, $p = 0.001$. Results of Bonferroni-corrected pairwise comparisons between groups are presented below.

#### 3.2.1. CIS patients vs. controls

##### 3.2.1.1. Memory and executive measures

The first set of analyses explored deviations of the average performance of CIS patients from age- and education-adjusted normative sample means. With respect to memory test scores, shown in Table 3, CIS patients as a group scored lower than controls on the following measures: immediate recall of WMS-III Story B (first trial) and Memory for Digits Forward. The patients' increased difficulty in memorizing Story B compared to Story A was reflected in significantly elevated Immediate and Delayed Differential Story Recall Indices.

With respect to the executive measures, Table 4 presents both raw values and standard scores, in order to facilitate comparisons. CIS patients showed elevated RTs on both conditions of the Inhibition task as compared to controls, and significantly reduced scores on the Inhibition Index for RT (RT on Change Trials minus RT on Baseline Trials). Error rates for the CIS group were slightly below average. In other words, although these patients tended to respond generally less rapidly than controls, they showed no evidence of increased difficulty in inhibiting the preponderant stimulus-response association. The tendency for elevated RTs was not evident on the Strategy Set-Shifting Task where, again, CIS patients as a group made fewer errors than controls. This difference reached significance for errors on Response Switching Trials (where participants were required to switch responding hand rather than cognitive strategy). This trend remained significant when controlling for error rate on Constant Trials, as indicated by the significantly lower than expected Response Switching Index values for Errors.

##### 3.2.1.2. Incidence of cognitive deficits among CIS patients

Next, we assessed the distribution of CIS patients displaying significantly reduced scores on a particu-

Table 2
Cognitive and emotional status (mean ± SD, range in parentheses)

| | RR-MS ($n = 49$) | CIS ($n = 44$) | CIS→CDMS ($n = 25$)[3] | Controls ($n = 230$) |
|---|---|---|---|---|
| CESD | 17.7 ± 13.5 (2–42) | 12.2 ± 11.1 (3–45) | 10.6 ± 9.3 (1–36) | – |
| STAI-A (State Anxiety) | 38.2 ± 11.1 (20–64) | 36.6 ± 12.4 (20–68) | 35.8 ± 11.6 (20–68) | – |
| STAI-B (Trait Anxiety) | 46.4 ± 11.5 (29–67) | 44.1 ± 10.3 (26–68) | 43.6 ± 10.6 (26–61) | – |
| Fatigue* | 36.4 ± 10.4 (12–51) | 30.9 ± 11.2 (10–50) | 32.1 ± 10.9 (10–50) | – |
| WASI Vocabulary (z) | 1.10 ± 0.96 (−0.94 to 3.1)[2] | 0.94 ± 0.99 (−1.5 to 3.0)[2] | 1.1 ± 1.2 (−1.5 to 3.1)[2] | 0.11 ± 1.0 (−1.5 to 3.1) |
| WASI Matrices (z) | −0.11 ± 0.8 (−2.1 to 1.2) | −0.18 ± 0.8 (−2.5 to 1.4) | −0.20 ± 0.9 (−2.5 to 1.4) | 0.09 ± 1.2 (−1.7 to 3.0) |
| Estimated IQ | 107.2 ± 10.5 (86–126) | 105.1 ± 9.4 (84–126) | 104.2 ± 9.6 (84–125) | 0.10 ± 1.1 (−1.6 to 2.9) |

*Significant RR-MS vs. CIS group differences ($p < 0.01$).
[1]Significantly different than 0 at $p < 0.0001$.
[2]Each patient group had higher raw scores than the control group ($p < 0.01$).
[3]Subgroup of CIS patients who, as of December 2010, fulfilled criteria for Clinically Definite MS (baseline information is presented here).
*Abbreviations:* CESD: Center for Epidemiological Studies Depression scale, STAI-A: State-Trait Anxiety Inventory Form Y, Fatigue: Fatigue Severity scale, WASI: Wechsler Abbreviated Scale of Intelligence.
Note: Anxiety, depression, and Fatigue scores not available on controls.

Table 3
Descriptive statistics on memory indices

| | RR-MS ($n = 49$) | | CIS ($n = 44$) | |
|---|---|---|---|---|
| | M ± SD | z | M ± SD | z |
| *Logical Memory Immediate recall (max score)* | | | | |
| LM-I-A (25) | 16.4 ± 3.3 | −0.07 ± 0.74 | 16.8 ± 3.7 | 0.07 ± 0.94 |
| LM-I-B1 (25) | 12.7 ± 4.2 | −0.73 ± 1.1† | 13.9 ± 3.1 | −0.38 ± 0.93* |
| LM-I-B2 (25) | 17.4 ± 3.9 | −0.64 ± 1.1† | 18.9 ± 3.6 | −0.16 ± 1.0 |
| Immediate Recall Index[1] | 29.1 ± 6.2 | −0.46 ± 0.8† | 30.8 ± 6.5 | −0.17 ± 0.90 |
| Learning Slope[2] | 4.7 ± 2.5 | 0.25 ± 0.91 | 4.9 ± 2.6 | 0.32 ± 1.0 |
| Differential Immediate Story Recall[3] | 3.7 ± 4.2 | 0.75 ± 1.2† | 2.9 ± 3.8 | 0.50 ± 1.0** |
| *Logical Memory Delayed recall (max score)* | | | | |
| LM-II-A (25) | 14.4 ± 3.8 | −0.08 ± 0.67 | 15.7 ± 4.4 | 0.24 ± 1.0 |
| LM-II-B (25) | 15.2 ± 4.7 | −0.49 ± 1.2** | 17.2 ± 4.0 | −0.11 ± 1.1 |
| Delayed Recall Index[4] | 30.8 ± 6.5 | −0.29 ± 0.81 | 33.4 ± 8.1 | −0.08 ± 1.1 |
| Retention Index Story A[5] | 91.9 ± 19.6 | 0.11 ± 1.3 | 95.7 ± 19.4 | 0.36 ± 1.2 |
| Retention Index Story B[6] | 91.5 ± 12.6 | 0.07 ± 0.9 | 91.9 ± 14.5 | 0.03 ± 1.0 |
| Recognition | 26.2 ± 2.3 | −0.10 ± 0.65 | 27.3 ± 2.4 | 0.23 ± 0.78 |
| Differential Delayed Story Recall[7] | −0.82 ± 3.7 | 0.36 ± 0.93* | −1.5 ± 3.9 | 0.39 ± 1.0* |
| *Memory for Digits (max score)* | | | | |
| Forward (16) | 8.7 ± 2.0 | −0.51 ± 0.90** | 8.9 ± 2.4 | −0.48 ± 1.2* |
| Reverse (14) | 6.2 ± 2.1 | −0.49 ± 0.91** | 6.6 ± 2.8 | −0.31 ± 1.2 |
| Memory for Digits-Total[8] (30) | 7.4 ± 1.9 | −0.53 ± 0.88† | 7.7 ± 2.3 | −0.38 ± 1.1 |
| *Taylor Complex Figure* | | | | |
| Coping (36) | 34.4 ± 1.4 | 0.09 ± 0.64 | 35.2 ± 0.5 | 0.39 ± 0.34 |
| Delayed Reproduction (36) | 11.4 ± 6.9 | −0.10 ± 1.1 | 12.2 ± 5.4 | 0.18 ± 1.0 |
| Retention Index[9] | 58.6 ± 22.3 | −0.13 ± 0.97 | 62.6 ± 19.1 | 0.02 ± 0.86 |

Significantly different from 0: $^{*}p < 0.01$, $^{**}p < 0.001$, $^{\dagger}p < 0.0001$.
[1]LM-I-A plus LM-I-B1 score, [2]LM-I-B2 score minus LM-I-B1 score, [3]LM-I-A minus LM-I-B1, [4] LM-II-A plus LM-II-B score, [5](LM-II-A / LM-I-A)*100, [6](LM-II-B / LM-I-B1)*100, [7] LM-II-A minus LM-II-B1, [8]Memory for Digits Forward plus Memory for Digits Reverse, [9](Delayed Reproduction / Coping) * 100.

Table 4
Descriptive statistics for executive task performance

| | RR-MS ($n = 49$) | | CIS ($n = 44$) | | Controls ($n = 230$) |
|---|---|---|---|---|---|
| | M ± SD | z | M ± SD | z | M ± SD |
| *Inhibition Task* | | | | | |
| Baseline-RT | 754 ± 255 | 1.2$^{\dagger}$ | 744 ± 264 | 1.0$^{**}$ | 606 ± 160 |
| Baseline-% Errors | 2.9 ± 3.4 | −0.15 | 2.4 ± 3.5 | −0.17 | 3.8 ± 4.9 |
| Change-RT | 1029 ± 281 | 0.88$^{\dagger}$ | 960 ± 254 | 0.47$^{*}$ | 878 ± 220 |
| Change-% Errors | 6.0 ± 6.0 | 0.04 | 6.0 ± 5.6 | −0.10 | 7.5 ± 6.4 |
| Inhibition Index-RT[1] | 41 ± 27 | −0.08 | 34 ± 21 | −0.39$^{*}$ | 47 ± 28 |
| Inhibition Index-Errors[2] | 3.1 ± 6.8 | 0.11 | 3.6 ± 5.9 | 0.01 | 3.7 ± 6.7 |
| *Strategy Set-Shifting Task* | | | | | |
| Constant Trials-RT | 740 ± 185 | 0.42 | 676 ± 146 | −0.05 | 710 ± 240 |
| Constant Trials-% Errors | 16 ± 11 | −0.22 | 15 ± 9 | −0.21 | 17 ± 11 |
| S & R Switching Trials-RT | 996 ± 285 | 0.51$^{*}$ | 917 ± 241 | 0.04 | 930 ± 330 |
| S & R Switching Trials-% Errors | 12 ± 9 | −0.38$^{**}$ | 12 ± 8 | −0.35 | 16 ± 9 |
| R Switching Trials-RT | 904 ± 295 | 0.32 | 779 ± 195 | 0.01 | 820 ± 300 |
| R Switching Trials-% Errors | 13 ± 10 | −0.63$^{\dagger}$ | 13 ± 10 | −0.50$^{**}$ | 18 ± 11 |
| S & R Switching Index-RT[3] | 23.5 ± 13.4 | 0.21 | 24.6 ± 11.7 | 0.18 | 21.8 ± 14.7 |
| S & R Switching Index-Errors[4] | −2.4 ± 8.4 | −0.16 | −1.5 ± 6.9 | −0.13 | 1.8 ± 7.2 |
| R Switching Index-RT[5] | 9.1 ± 13.8 | 0.26 | 13.9 ± 12.5 | 0.11 | 11.6 ± 13.0 |
| R Switching Index-Errors[6] | −3.3 ± 9.8 | −0.50 | −2.4 ± 8.8 | −0.32$^{**}$ | 1.1 ± 0.09 |

Significantly different than 0: $^{*}p < 0.01$, $^{**}p < 0.001$, $^{\dagger}p < 0.0001$.
For all Group main effects, $p > 0.2$. Controls belonged to a subgroup of 230 neurologically intact volunteers chosen from the normative sample, matched on the distribution of gender, age, and education to the groups of patients.
[1]Percent increase in RT between Baseline and Change Conditions (smaller raw values indicate higher ability to inhibit the learned stimulus-response association and adopt a new one).
[2]Difference in error rate between Baseline and Change in percentage points.
[3]Percent increase in RT on trials requiring a switch in both strategy and response key (left-right; S & R Trials) compared to Constant trials (smaller raw values indicate higher switching ability).
[4]Difference in error rate on S & R trials compared to Constant trials (smaller raw values indicate higher switching ability).
[5]Percent increase in RT on trials requiring a switch in response key but not in strategy (R Trials) compared to Constant trials (smaller raw values indicate higher switching ability).
[6]Difference in error rate on R trials compared to Constant trials (smaller raw values indicate higher switching ability).

lar performance index compared to controls. "Poor" scores were considered those associated with a z score $< -1.5$ (with the exception of the Differential Story Recall indices were the cut-off was set to $z = +1.5$). As shown in Table 5, the proportions of CIS patients with very poor scores were significantly (by an order of 2 to 5) higher than the corresponding proportions in the normative sample on the following measures: Immediate Recall of the WMS-III Story B (first and second trial), and Differential Immediate Story Recall indices from the Logical Memory subtest.

A closer look at the distribution of CIS patients with deficits in two or more memory domains (i.e., short-term/working memory and secondary verbal memory, short-term/working memory and secondary non-verbal memory, or secondary verbal memory and secondary non-verbal memory) revealed significantly higher (18.2%) than the expected population rates (2.2% in the age-, gender-, and education-level-matched comparison sample; $X^2$, $p = 0.0001$). Conversely, the proportion of CIS patients (9.1%) with significantly impaired performance on two or more executive measures did not differ from the corresponding proportion in the normative sample (4.3%). The proportion of CIS patients who showed parallel deficits on one or more memory domains and on one or more executive measures was 4.5% as compared to 3.2% in the normative sample ($p > 0.3$).

### 3.3. Clinical variables and cognitive status among CIS patients

Correlations between clinical/psychoemotional (Pearson r for duration of illness, depression, anxiety, and fatigue scores and Spearman's rho for EDSS) and cognitive variables did not reveal significant associations (evaluated at $\alpha = 0.05/21 = 0.0023$ to control for Family-wise Type I error). There were only

Table 5
Percentage of patients and controls with scores in the impaired range (with a cutoff of 1.5 SDs)

| | RR-MS ($n = 49$) | CIS ($n = 44$) | Controls ($n = 230$) |
|---|---|---|---|
| *Memory* | | | |
| Memory for Digits Forward | 10.5** | 6.8 | 3.6 |
| LM-I-B1 | 22.9** | 11.5* | 4.9 |
| LM-I-B2 | 19.1** | 13.6* | 5.4 |
| Differential Immediate Story Recall[1] | 21.2** | 18.2** | 5.8 |
| LM-II-B | 14.6** | 4.5 | 4.0 |
| Differential Delayed Story Recall[2] | 10.5* | 6.8 | 4.5 |
| *Executive* | | | |
| Inhibition[3] | 10.6 | 7.5 | 9.2 |
| Switching[4] | 10.0 | 10.0 | 8.6 |
| *WASI* | | | |
| Vocabulary | 0.0 | 3.1 | 1.8 |
| Matrices | 6.5 | 9.1 | 3.5 |

[1]LM-I Story A minus Story B1 score, [2]LM-II Story A minus Story B score, [3]Inhibition Index-Errors, [4]Average of Shifting Index-Errors and Response Shifting Index-Errors (a deficit was defined as an average z score $< -1.5$ only when the average z score for corresponding Switching RT Indices was $> -0.05$ in order to avoid including individuals who showed significant increases in error rates coupled with significant decreases in RTs).
Significantly higher proportions for each patient group vs. controls: $^{*}p < 0.05$, $^{**}p < 0.01$.

non-significant trends indicating reduced performance with longer duration of illness for Memory for Digits-Forward ($r = -0.40, p = 0.008$), Strategy & Response Switching Trials-RT ($r = 0.37$, $p = 0.02$), Response Switching Index-RT ($r = 0.40, p = 0.01$), and Strategy and Response Switching Index-Errors ($r = 0.44$, $p = 0.006$). Complementary group-level analyses, contrasted CIS patients who presented with two or more memory deficits ($n = 9$) with the CIS patients ($n = 35$) who did not show impaired performance on any of the tests used in the present study. The dependent variables in these analyses included demographic (age, education, gender), clinical (i.e., number and type of clinical symptoms at the initial diagnosis of CIS, medication [treatment vs. no treatment], EDSS, disease duration), and psychoemotional measures (Fatigue scale, CESD, and STAI scores). No significant subgroup effects were noted with the exception of a significant tendency for non-impaired patients to score higher on the WASI Vocabulary subtest, $F(1,42) = 6.04, p = 0.018$. Similarly, analyses failed to reveal any differences on psychoemotional or cognitive measures between CIS patients with monofocal vs. multifocal involvement ($p > 0.5$). The small number of patients treated with Natalizumab did not permit formal comparison with the remaining RR-MS patients. A two-way ANOVA with treatment (yes/no) and patient group (CIS/RR-MS) did not reveal main effects or interactions involving treatment.

### 3.4. *Cognitive status and disease progression among CIS patients*

We did not find significant differences on any clinical, demographic, or cognitive variable ($p > 0.3$) between CIS patients who showed no disease progression ($n = 19$) within three years from the initial neuropsychological evaluation as compared to those who progressed to clinically definite MS ($n = 25$) during this time.

### 3.5. *MRI findings*

Analysis of brain imaging data indicated that periventricular white matter lesions in the CIS group was by far the most frequent finding, occurring in all but one of the CIS patients (Table 6). No significant correlations were found between the presence of lesion(s) in each of the predefined brain regions shown in Table 6 and the cognitive or psychoemotional measures evaluated.

### 3.6. *Comparison of CIS and RR-MS patients*

Raw and z scores on the cognitive measures for the RR-MS group are shown in Tables 3 and 4. On a group basis, RR-MS patients performed significantly lower than CIS patients on only two measures: Immediate

Table 6
Distribution of lesion location among patients with CIS ($n = 43$)

| # of lesions | | | |
|---|---|---|---|
| Periventricular | 41 | # Supratentorial | |
| Corona radiata | 34 | Mean | 15.5 |
| Semioval center | 28 | Median | 13.0 |
| Subcortical s/tentorial | 30 | SD | 11.5 |
| Corpus callosum | 17 | Range | 1–44 |
| Thalami | 12 | | |
| Basal Tanglia | 3 | | |
| Cerebellum | 9 | # Infratentorial | |
| Cerebellar Peduncles | 13 | Mean | 1.7 |
| Pons | 10 | Median | 1.0 |
| Midbrain | 9 | SD | 2.7 |
| Medulla | 3 | Range | 0–13 |

Recall of Logical Memory Story B (second trial only) and Logical Memory Recognition Index ($p > 0.05$). However, these differences were eliminated when controlling for duration of illness. The incidence of cognitive deficits was not significantly different between CIS and RR-MS groups on specific indices (see Table 5). The proportions of patients with deficits on two or more memory domains (18.2 vs. 19.1%, respectively) were comparable to those with deficits on two or more executive measures (4.1 vs. 9.1%).

## 4. Discussion

The present study investigated primary and secondary memory and motor/executive performance of patients with CIS in reference to age- and education-adjusted Greek population norms and in comparison to patients with relapsing-remitting MS selected from a larger clinical cohort for displaying mild physical disability. Moreover, clinical, psychoemotional and radiologic variables were examined as potential correlates of initial cognitive status as well as of CIS progression to clinically definite MS. The main finding here was that performance of CIS patients deviated from age- and education-adjusted normative scores on measures of verbal short-term and secondary memory. Specifically, these patients showed reduced performance on the Memory for Digits-Forward, and increased relative difficulty for both immediate and delayed recall of the second of two consecutive passages in the Logical Memory WMS-III subtest. This pattern of memory performance was very similar to the profile displayed by RR-MS patients (see also [48]).

### 4.1. Memory performance

The observed impairment in verbal short term memory is rather straightforward to interpret, as it most likely reflects difficulty in maintaining semantically and phonologically unrelated information in transient storage. The process of maintaining active phonological representations in consciousness and/or the covert rehearsal mechanism may be responsible for the reduced performance on this task (for a review see [62]).

On the other hand, the increased relative difficulty in recalling items from Story B on the Logical Memory scale is more difficult to interpret. This task is complicated by the fact that the first attempt to memorize Story B in the Greek version of the Logical Memory subtest proved to be slightly more difficult, on average, than memorization of Story A in the normative data set. However, examination of age- and education level-adjusted standard scores revealed that the relative difficulty, especially in the immediate recall of Story B, was clearly evident at the individual level, with 3–5 times as many CIS patients showing this trend than persons in the normative sample. The patients' poor memory performance did not reflect a general decline in intellectual ability, as the percentage of patients with lower than expected performance on intelligence measures was not different as compared to the normative sample.

The pattern of patient performance on the Logical Memory subtest likely rules out a number of potential interpretations. Thus, CIS patients, similar to the control participants, were able to benefit from repeated presentation and recall of story B (as indicated by typical learning indices) and were able to retain information from immediate recall to the 30-minute delayed recall (as indicated by typical retention indices). Therefore, both learning through repeated exposure and recall as well as the consolidation processes appeared to be intact. It is, also, notable that relative, delayed recall ability of story B was, on average, impaired equally to the immediate recall ability (although the proportion of patients who showed significantly impaired Delayed Differential Recall indices was not significantly elevated). This may imply a mitigating tendency for the degree of relative recall difficulty of story B following the 30 min delay. It is possible that active semantic reorganization processes, operating during memory consolidation, may have partially compensated for the difficulties in the initial encoding and short-term maintenance of story B elements.

Alternative accounts include increased susceptibility to proactive interference (associated with memorization of story A), and impaired verbal short-term and/or working memory. The latter could directly impact on the ability to memorize final story elements leading to lower total recall scores. These hypotheses have been explored by Panou et al. [47] revealing that, although there was no evidence of increased susceptibility to proactive interference, a reduced immediate recall of Story B was found that was largely due to difficulty in memorizing elements from the middle and final portions of the story. In agreement with prevailing notions, the ability to memorize final elements from a passage or word list depends largely on primary memory processes and/or on the ability to maintain temporal order information [65]. It remains to be seen if the same pattern of serial-position effects is present among CIS patients. In one of the first studies on the early stages of definite MS, short-term retention efficiency seemed to be particularly vulnerable to the effects of proactive interference and presumed to reflect difficulties in the initial encoding of information [26]. Short-term memory difficulties correlated reasonably well with the number of years since disease onset.

### 4.2. Performance on motor/executive tasks

CIS patients showed no evidence of increased difficulty in inhibiting a preponderant strategy and adopting a different stimulus-response mapping, as indicated by equal to or lower than expected error rates and better than expected performance index values on the Inhibition task. Nevertheless, they tended to respond less rapidly than controls on both conditions of the task (Baseline & Change trials). In other words, they showed typical signs of a reversed speed-accuracy trade-off, suggesting increased caution while performing this task. On the Strategy Set-Shifting task, CIS patients displayed similar average RTs and, if anything, slightly lower error rates than controls. The lack of evidence for impaired executive functions is consistent with patient performance on the only other task used in the present study, considered to assess executive function, namely Memory for Digits-Reverse (despite their significant impairment in verbal short-term memory).

### 4.3. CIS vs. MS patients

While there have been reports of deficits for both primary and secondary memory functions in CIS patients [8,16–18,24,37], this is the first study to directly compare CIS patients with a closely matched group of patients with established relapsing-remitting MS. On a group basis, CIS and RR-MS patients displayed similar degree of difficulty on the immediate recall of Story B and increased relative difficulty on both the immediate and delayed recall of Story B as compared to Story A. In addition, both groups showed reduced performance on a measure of short-term verbal memory. These trends were also evidenced at the individual level, where nearly one in five patients in either group showed significantly impaired performance on at least two memory domains (primary or secondary, verbal or non-verbal). RR-MS patients showed evidence for a greater degree of impairment, both on average and at the individual level, on story B recall (both immediate and delayed), in addition to the Differential Recall Indices, and Memory for Digits-Reverse. In addition, they showed increased RT on both executive tasks (yet unaffected accuracy rates and executive indices), consistent with a more general processing speed deficit. In agreement with this result, De Sonneville et al. [13] reported greater relative impairment in processing speed than accuracy among MS patients. Moreover, Landro et al. [34] found slowed information processing speed and impaired working memory, in the presence of unimpaired executive functions, a pattern similar to what we found in the present study.

The similarities in memory performance between the two groups of MS patients evaluated are made all the more important in view of the fact that our group of RR-MS patients, despite showing relatively mild physical disability (as indicated by EDSS scores $\leqslant 3$), included a higher percentage of individuals with CESD scores indicative of symptoms of depression and higher levels of reported fatigue than the CIS group. It should be noted, however, that the percentage of patients who reported potentially clinically significant levels of depression, anxiety, and fatigue was all but negligible in the CIS group. This is in accordance with the baseline anxiety and depression rates reported by Di Legge et al. [14] and suggests that, despite very mild physical disability and independently of gender and age at clinical onset, the disease may already impact psychological well-being. Mood changes in the early stages of the disease may be caused in some cases, or exacerbated in others, by uncertainly about diagnosis and prognosis. This concept was raised among others by O' Connor et al. [45] who, in a follow-up study on patients with suspected MS, noted that distress over diagnostic uncertainty decreased significantly when the patients were given a definite diagnosis, irrespective of the diagnostic outcome. Furthermore, the degree of social stress experienced by CIS patients is closely linked to the emergence of psychiatric symptoms [36]. Unless the progression of neurological symptoms and physical disability in CIS patients is studied longitudinally over a sufficiently long period, it is difficult to ascertain the degree to which the putative cognitive impairments can be attributed to the demyelinating processes or to the psychological burden brought by the disease.

### *4.4. Correlates of memory deficits and of disease progression*

The present study revealed that 8% (2/25) of our CIS patients disease duration of $\leqslant$ 1 year suffered of one of more relapses, whereas 35.7% (15/42) of those with disease duration of $\leqslant$ 5 years had experienced such relapses, thus meeting Poser's criteria [54] for conversion to clinically definite MS. These conversion rates are somewhat lower than those reported previously for natural-history cohorts [19] or untreated CIS patient groups participating in clinical trials [9,28,29], and are consistent with the thesis that MS in Crete follows a more benign course than elsewhere [38]. However, the median time to progression to CDMS found in our study (2.3 years; range 0.8–5.4 years) is comparable to that reported for the UK (2.0 years; Brex, 2002), Lyon, France (1.9 years; [10]), and Gothenburg, Sweden CIS cohorts (3.25 years; [15]).

As indicated above, we found no differences in the psychoemotional, neuropsychological, or MRI variables studied between the CIS patients who showed no evidence (clinical or radiological) for disease progression as compared CIS patients who converted to clinically definite MS). It is presently unclear if this relates to the relatively short time that elapsed between neuropsychological assessment and CDMS conversion. It should be stressed that examination of more sensitive indices of structural or functional anomalies (including global and regional atrophy, white matter damage, and regional cerebral perfusion; [2,19,55,66]) may have provided better correlates of cognitive functi on if they were included in the analyses. Moreover, additional or complementary measures of cognitive function, such as those provided by widely used neuropsychological assessment batteries for MS (such as the brief repeatable battery of neuropsychological tests; [5,52, 57]) may have revealed additional key deficits among CIS patients (as for instance in processing speed and attention).

The course and evolution of cognitive performance in early MS remains controversial, as some authors consider CIS to be the neurological and cognitive precursor of MS [1,67]. Clear evidence of cognitive decline in CIS has been difficult to procure empirically. For instance, in the longitudinal study of Feinstein et al. [16], attention deficits documented at baseline assessment remained unchanged in severity in patients who did not present with clinical relapses at a four-year follow up. Deterioration of auditory attention and of immediate and delayed recall ability was observed among patients who developed a chronic progressive course. Conversely, there was significant overlap in cognitive performance between patients who later developed a relapsing-remitting course of MS and CIS patients, in agreement with our data. Consistent with the notion of progressive deterioration in cognitive function in CIS are the moderate correlations between illness duration and measures of short-term memory and executive function (mainly strategy and/or response switching ability). Among the clinical and general cognitive factors which were evaluated as potential predictors of a cognitively "benign" course of the disease, only a measure of crystallized verbal ability appeared to play a significant role. This finding is consistent with the notion of cognitive reserve and its role as a positive prognostic indicator for cognitive decline in progressive neurological disorders [7,35].

One of the clinical variables that may affect cognition in the long-term concerns early versus delayed immunomodulatory treatment in patients with CIS. For instance, Kappos et al. [29,30] suggested that treatment with interferon beta-1b in patients with a first clinical event may have delayed conversion to clinically definite MS. Treatment was particularly beneficial in patients with less active or disseminated disease. Other studies have reported significant longitudinal improvements in cognitive function in patients with active RR-MS who, at baseline, were cognitively impaired [39, 49]. The above observations are in line with the hypothesis that inhibition of inflammation and lesion development with immunomodulatory agents may preserve cognitive function. It should be noted, however, that only 7 patients in the current group of CIS patients were under INF treatment precluding formal analyses of treatment effects on clinical or cognitive progression.

To conclude, our results suggest the presence of verbal short-term memory deficits in a substantial proportion of CIS patients and that these findings were similar to those detected in patients with RR-MS, implying that a common pathophysiological mechanism related to the demyelinating process is operational. However, it is considerably more difficult to venture into an interpretation of the disproportionate impairment in recall of the second of two sequentially administered story passages. Inspection of individual standard test scores highlights the presence of marked individual variability in the cognitive profile of the presumed impairments. At least in part, this variability may be due to measurement variability in time, an issue that awaits further investigation along with the long-term predictive value of early signs of cognitive impairment in CIS.

## Acknowledgments

This Research Project was partially supported by the Association for Research and Treatment of Neurologic Disorders of Crete ("EY ZHN"), which is philanthropic (non-profit) organization that supports the clinical and research activities of the Neurology Department of the University Hospital of Crete.

## Potential conflicts of interest

Nothing to report.
